# Winner and loser effects are modulated by hormonal states

**DOI:** 10.1186/1742-9994-10-6

**Published:** 2013-02-11

**Authors:** Ryan L Earley, Chung-Kai Lu, I-Han Lee, Stephanie C Wong, Yuying Hsu

**Affiliations:** 1 Department of Biological Sciences, University of Alabama, Box 870344, 300 Hackberry Lane, Tuscaloosa, AL, 35401, USA; 2 Department of Life Science, National Taiwan Normal University, No. 88, Section 4, Ting-Chou Rd, Taipei 11677, TAIWAN

**Keywords:** Animal contest, Information, Winner-loser effect, Cortisol, 11-ketotestosterone, Testosterone, Oestradiol, *Kryptolebias marmoratus*

## Abstract

**Introduction:**

Many animals use information acquired from recent experiences to modify their responses to new situations. Animals’ decisions in contests also depend on their previous experience: after recent victories individuals tend to behave more aggressively and after defeats more submissively. Although these winner and/or loser effects have been reported for animals of different taxa, they have only recently been shown to be flexible traits, which can be influenced by extrinsic factors. In a mangrove killifish (*Kryptolebias marmoratus*), for instance, individuals which lost an earlier contest were more likely than others to alter contest decisions after a recent win/loss. This result suggests that individuals perceiving themselves to have worse fighting abilities are more inclined to adjust contest strategy based on new information. If this is the case, an individual’s propensity to modify behaviour after a win/loss might also be modulated by intrinsic mechanisms related to its ability to fight. Stress and sex steroid hormones are often associated with an individual’s contest behaviour and performance, so, in this study, we tested the hypothesis that an individual’s propensity to change behaviour after wins or losses also depends on its hormonal state.

**Results:**

Our results show that an individual’s propensity to adjust contest decisions after wins and losses does depend on its hormonal state: individuals with lower levels of cortisol (F), testosterone (T) and 11-ketotestosterone (KT) are more receptive than others to the influence of recent contest experiences, especially losing experiences, and the influences last longer. Furthermore, although winning and losing experiences resulted in significant changes in behaviour, they did not bring about a significant change in the levels of F, T, KT or oestradiol (E2).

**Conclusions:**

This study shows that an individual’s receptivity to the influence of recent wins and losses is modulated by its internal state, as well as by extrinsic factors. Individuals with hormonal profiles corresponding to lower aggressiveness and a reduced likelihood of winning were more likely to alter contest decisions after a recent win/loss. The results also suggest that F, T, KT and E2 are not the primary physiological mechanisms mediating winner-loser effects in this fish.

## Introduction

Many animals modify behavioural decisions based on previous experiences. Female field crickets (*Teleogryllus oceanicus*), for instance, are more choosy after interacting with an attractive male (they mount subsequent males more slowly and retain males’ spermatophores for less time), but less choosy after interacting with an unattractive male [1]. Least chipmunks (*Tamias minimus*) and golden-mantled ground squirrels (*Spermophilus lateralis*) have a higher probability of visiting a patch in which they found seeds previously than a patch where they did not find seeds; their preferences for different patches depend on the combined results of multiple visits [2]. These prior experiences are thought to provide individuals with information about the quality of available mates and of different food patches, respectively, which influences the individuals’ subsequent behavioural decisions.

Animals’ decisions in contests also are influenced by their previous experiences: individuals that have recently won tend to behave more aggressively towards a new opponent and enjoy an elevated chance of winning again (winner effect) while individuals that have lost recently tend to behave more submissively and suffer a higher chance of losing again (loser effect) [3,4]. The outcomes of previous contests probably provide individuals with sampling information about how their fighting ability compares with those of others in the population, which in turn influences their subsequent contest decisions [5-7]. Winner and/or loser effects have been observed in a wide range of taxa and are usually reported as a species-specific characteristic (i.e., some species exhibit winner and/or loser effects while others do not). It is only recently that an individual’s propensity to alter contest decisions after acquiring winning and/or losing experiences has been shown to be a flexible trait, modulated by extrinsic factors. For instance, in California mice (*Peromyscus californicus*), only individuals that acquired winning experiences in their home cages, and not those that acquired experiences in unfamiliar cages, displayed winner effects [8]. The information from previous wins in their own territories, where the benefits associated with victory are high, thus appears to have extra value to individuals of California mice, a territorial species. The lack of a winner effect when individuals are not in their own territories might enable individuals to avoid paying the costs of aggressive interactions when the benefits associated with victory are low [8].

The fact that the propensity to modify contest decisions differs between individuals exposed to different circumstances suggests not only that information from recent wins/losses differs in its value to individuals in different circumstances but also that individuals monitor their circumstances in determining whether and how to use the information in future contest decisions. In *Kryptolebias marmoratus*, a mangrove killifish, the outcomes of fights experienced one month previously influence how individuals respond to a win or loss one month later; individuals that were given a forced losing experience one month previously were more receptive to the influence of a recent contest experience (i.e., exhibited greater changes in contest behaviour) than those that received a forced winning experience [9]. Information is useful to an individual to the extent that it helps the individual’s decision making by reducing its uncertainty [10]. The findings in *K*. *marmoratus* therefore indicate that individuals perceiving themselves to have worse fighting abilities (as a result of the forced losing experience one month previously) had a higher degree of uncertainty in how to respond to a new competitor one month later than those perceiving themselves to have better fighting abilities. A small scale capture-recapture study in this fish suggests a high population turnover - only two out of 14 marked fish were recaptured among the 81 fish caught over the subsequent 5 days [11]. Therefore, an experience from one month ago may still provide an individual with some general information about its fighting ability, but be of limited use if it does not relate to the fighting ability of the individuals currently in the local population. Without updated information on this, individuals perceiving themselves to have poor fighting abilities would face an uncertain cost of engaging in a new contest, ranging from low (when the local population is composed of a high proportion of weak individuals) to high (conversely). The cost for individuals perceiving themselves to have good fighting abilities, however, is probably less variable, ranging only from low (weak local population) to moderate (strong local population). The greater uncertainty in the cost of in engaging in a new contest may therefore prompt individuals with low perceived fighting abilities to be more attentive to information from a recent experience. If this is the case, it is probable that an individual’s propensity to use the information also depends on intrinsic factors correlated with its fighting ability.

Steroid hormones, especially glucocorticoids, androgens and oestrogens, have long been shown to be closely associated with an individual’s aggressive behaviour and dominance ability [12-15]. Glucocorticoids are associated with stress and appear to have complex relationships with an individual’s dominance status [14,16]. Studies using captive animals often find that individuals with higher levels of glucocorticoids or which produce more glucocorticoids in response to stress tend to exhibit lower levels of aggression and are less capable of attaining dominant status [17,18]. Data from field studies of animals living in groups, on the other hand, frequently report dominant individuals to have higher levels of glucocorticoids [19-21]. Testosterone has been linked to dominance and to increases in aggressive behaviour [14]. For instance, in *K*. *marmoratus*, individuals with higher levels of endogenous testosterone are more aggressive [22], quicker to attack and have a higher chance of winning [23]. 11-ketotestosterone, a potent androgen in fish, can also facilitate aggressive behaviour; for instance, its level is higher in dominant male cichlids (*Oreochromis mossambicus*; *Neolamprologus pulcher*) [24,25]. Oestrogen is also found to increase the probability that birds and rodents engage in aggressive behaviour and the intensity of the aggression, although it reduces aggression in California mice (see [15] for a review).

In this study, we investigated whether endocrine status modulates winner and loser effects. More specifically, we examined in the mangrove killifish whether and how an individual’s levels of cortisol (F), testosterone (T), 11-ketotestosterone (KT) and oestradiol (E2) influence its propensity to alter contest decisions after exposure to winning and losing experiences. We investigated the relationship between hormone status and both the magnitude and the longevity of winner and loser effects using a 3 (experience treatments) × 3 (time-decay treatments) factorial design. In the experiment, each of 270 focal fish was given three consecutive winning, losing or no-contest experiences (randomly allocated, and referred to as EW, EL and EN respectively) and then fought with a sized-matched naïve opponent. (Fish given no-contest experiences were handled in the same way as others, except that they did not face an opponent in their three training sessions.) One third of these size-matched contests took place on the day of the final experience training, one third after a delay of one day and one third after a delay of seven days (again chosen at random and referred to as 0d, 1d and 7d treatments respectively). Hormone samples were collected before the fishes’ experience training (pre-experience levels), and both before (pre-contest levels) and after (post-contest levels) their sized-matched contests, in each case by isolating the fish in a beaker of water and subsequently analysing the water. The study animal, experimental design and procedures and the statistical analyses are all explained in ‘Materials and Methods’ below.

If subordinate individuals are influenced to a greater extent by recent wins and losses, we would expect individuals with lower levels of T, KT and E2 to display stronger winner and/or loser effects, since the levels of these hormones are frequently lower in individuals that behave submissively. The relationship between cortisol levels and the importance of the experience effects in this fish is more difficult to predict as studies that investigated the relationships between F levels and dominance and/or aggressiveness have produced mixed results as discussed earlier. A recent study of *K*. *marmoratus*, however, showed F levels to be positively correlated with T levels and aggressiveness [22]. We therefore expected individuals with lower F levels to display stronger winner and/or loser effects.

## Results

### Influence of contest experience × time-decay on pre-contest hormones

**Figure 1:**
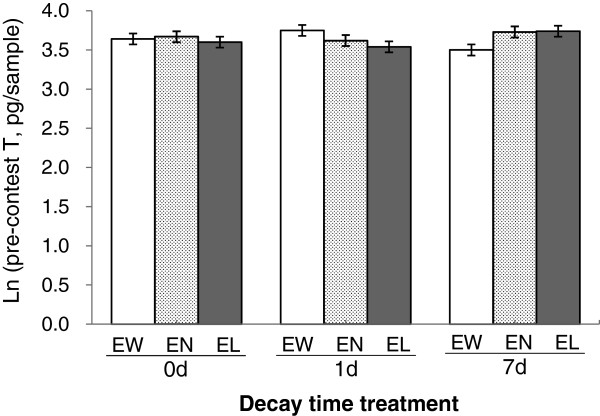
**Pre**-**contest T levels for the focal individuals assigned to different contest experience** × **time**-**decay treatments.** Means (± SE) are least squares means adjusted for pre-experience T level and strain type. Within each of the time-decay treatments, none of the pair-wise differences between different experience treatments reached significance (Tukey multiple comparisons, all *P* > 0.05).

Neither contest-experience nor time-decay treatments had a significant main effect on the levels of F, T, KT or E2 (Table 1). The interaction between the two treatments, however, did significantly affect pre-contest T. For the 1d decay-time treatment, pre-contest T was the highest in the EW individuals, followed by EN and EL individuals (EW > EN > EL); however, the relationship in T levels was reversed for the 7d time-decay treatment (EW < EN < EL) (Figure 1), although none of the pair-wise differences for any of the time-decay treatments reached significance (P > 0.050, Tukey multiple comparisons). There was very little difference in pre-contest T levels between the experience groups in the 0 time-decay treatment. The effects of the interaction between the two types of treatments on F, KT and E2 were not significant.

**Table 1:** The effect of contest experience × time-decay treatments on pre-contest hormones

		**Pre-contest F**			**Pre-contest T**			**Pre-contest KT**			**Pre-contest E2**		
**Variable**	***df***	***b ±SE***	***F***	***P***	***b ±SE***	***F***	***P***	***b ±SE***	***F***	***P***	***b ±SE***	***F***	***P***
Experience	2		1.72	0.181		0.41	0.663		0.13	0.878		1.40	0.248
Time-decay	2		2.36	0.097		0.07	0.935		0.03	0.968		1.09	0.338
Exp×Time	4		1.17	0.324		2.77	0.028*		1.24	0.294		0.70	0.591
Pre-Exp level	1	0.29 ± 0.06	22.42	<0.001*	0.61 ± 0.06	118.22	<0.001*	0.60 ± 0.05	162.31	<0.001*	0.46±0.06	57.98	<0.001*
Strain	4		3.52	0.008*		2.11	0.081		0.75	0.560		0.82	0.511

Multiple linear regression modelling the importance of contest experience × time-decay treatments on pre-contest hormones, controlling for the corresponding pre-experience hormone levels and strain type (*N* = 270, *df*: numerator degree of freedom, *: *P* ≤ 0.05, Exp: Experience, Time: Time-decay).

### Winner/loser effects and the importance of hormonal state

**Figure 2:**
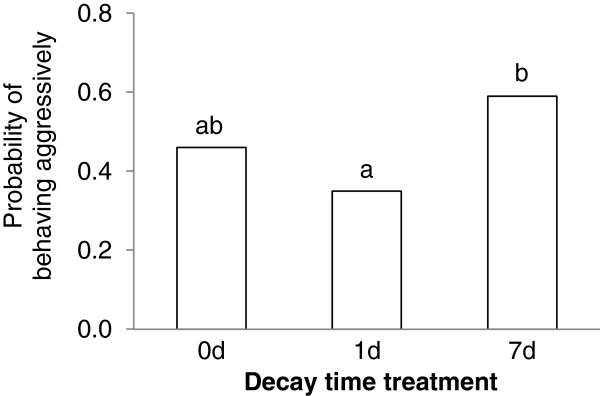
**The likelihood of behaving aggressively for the focal individuals assigned to different decay-time treatments.** Bars labelled with different letters differ significantly in the likelihood (P < 0.05, likelihood ratio χ^2^ test).

Contest experience significantly affected the likelihood that focal individuals would behave aggressively in, and win, the size-matched contests (Table 2). A focal individual was deemed to be aggressive if it initiated attacks or retaliated with attacks when attacked. When analysed separately, winning and losing experiences had opposite effects (positive and negative, respectively) on the contest behaviours exhibited during size-matched contests, although the positive effect of winning experiences on winning probability did not reach significance. The probability that focal individuals would behave aggressively in the size-matched contests depended significantly on the time-decay treatment, but there was no significant interaction with experience treatment. Further analyses showed that focal individuals assigned to the 7d time-decay treatment (taking part in size-matched contests seven days after the completion of their experience training) were more likely to behave aggressively than those assigned to the 1d time-decay treatment (LR χ^2^_1_ = 8.74, P = 0.003), while focal individuals assigned to the 0d time-decay treatment did not differ significantly from the 1d or 7d treatments (as shown in Table 2) (Figure 2).

**Table 2:** The influence of contest experience on contest behaviours and its dependence on hormonal states

		**Behaving aggressively**			**Winning contests**		
***Variable***	***df***	***b ±SE***	***LRχ***^***2***^	***P***	***b ±SE***	***LRχ***^***2***^	***P***
Experience	2		43.56	<0.001*		17.70	<0.001*
Win^1^	1	1.06 ± 0.42	6.98	0.008*	0.52 ± 0.31	2.87	0.093
Lose^1^	1	-1.74 ± 0.57	10.13	0.002*	-1.85 ± 0.34	6.49	0.011*
Time-decay	2		8.84	0.012*		1.66	0.437
1d^2^	1	-0.65 ± 0.47	2.02	0.156	0.22 ± 0.34	0.42	0.518
7d^2^	1	0.08 ± 0.51	0.03	0.868	0.42 ± 0.33	1.66	0.198
Exp×Time	4		5.55	0.236		7.08	0.132
Strain	4		5.35	0.253		12.69	0.013*
Pre-Cont F	1	0.13 ± 0.20	0.45	0.503	-0.06 ± 0.13	0.18	0.669
Exp×Pre-ContF	2		9.65	0.008*		1.03	0.598
Time×Pre-Cont F	2		4.63	0.099		1.95	0.378
Exp×Time×Pre-Cont F	4		13.85	0.008*		2.80	0.592
Pre-Cont T	1	0.43 ± 0.43	1.07	0.302	0.05 ± 0.34	0.02	0.880
Exp×Pre-Cont T	2		9.89	0.007*		1.73	0.421
Time×Pre-Cont T	2		3.33	0.190		5.02	0.081
Exp×Time×Pre-Cont T	4		11.32	0.023*		13.46	0.009*
Pre-Cont KT	1	1.41 ± 0.66	4.95	0.026*	1.59 ± 0.72	6.14	0.013*
Exp×Pre-Cont KT	2		10.60	0.005*		9.68	0.008*
Time×Pre-Cont KT	2		12.42	0.002*		5.76	0.056
Exp×Time×Pre-Cont KT	4		5.78	0.216		11.62	0.020*
Pre-Cont E2	1	0.03 ± 0.24	0.02	0.897	-0.05 ± 0.20	0.06	0.813
Exp×Pre-Cont E2	2		2.13	0.345		0.83	0.661
Time×Pre-Cont E2	2		2.87	0.239		2.35	0.309
Exp×Time×Pre-Cont E2	4		9.11	0.058		9.28	0.054

Multiple logistic regression modelling the influence of contest experience on the probabilities of behaving aggressively and winning contests and the degree to which these influences depended on hormonal states. The focal individuals’ F, T, KT and E2 levels were correlated with each other, so, to avoid multicolinearity problems, each of the hormones (F, T, KT and E2) was tested separately. The first section of the table shows the models that tested the importance of contest experience, decay-time treatment and the interaction between them on contest behaviour, controlling for strain type. The second to the fifth sections of the table show the effects of pre-contest F, T, KT and E2, respectively, and their interactions with contest experience and time-decay treatments, with the variables in the first section included in the models. (*N* = 270, *LRχ*^*2*^: likelihood ratio *χ*^*2*^, *: *P* ≤ 0.05, Exp: Experience, Time: Time-decay, Cont: Contest).

^1^indicator variables, individuals with no recent experience form the baseline group.

^2^indicator variables, 0d decay time treatment is the baseline group.

We used two sets of logistic regression models to see how the likelihood that the focal fishes would behave aggressively in and win the size-matched contests depended on their hormone levels and experience × time-decay treatment. The simpler models used pre-contest hormone levels as one of their explanatory variables (Table 2); the more complex models broke pre-contest hormone levels down into pre-experience hormone levels and the difference between pre-contest and pre-experience levels (Addition file 1). The more complex models were not significantly better (likelihood ratio tests) than the simpler models in explaining either the likelihood of behaving aggressively (F: χ^*2*^_*9*_ = 8.82, *P* = 0.454; T: χ^*2*^_*9*_ = 9.04, *P* = 0.434; KT: *χ*^*2*^_*9*_ = 4.38, *P* = 0.885; E2: *χ*^*2*^_*9*_ = 7.28; *P* = 0.608) or the likelihood of winning (F: *χ*^*2*^_*9*_ = 8.20, *P* = 0.514; T: *χ*^*2*^_*9*_ = 4.00, *P* = 0.911; KT: *χ*^*2*^_*9*_ = 7.88, *P* = 0.546; E2: *χ*^*2*^_*9*_ = 4.70; *P* = 0.860). Since these results indicate that breaking hormone levels down into pre experience + change does not lead to a significant improvement, we focus the rest of the analysis of the results on the simpler models using only pre-contest hormones, but an examination of table 2 and Additional file 1 will show that the conclusions would be the same if the more complicated models were used and that both pre-experience hormone levels and the change from pre-experience to pre-contest hormone levels worked in the same direction.

The extent to which contest experience influenced the focal individuals’ probability of behaving aggressively and probability of winning were both dependent on the focal individual’s F, T and KT levels; many of the interactions between pre-contest hormones, contest experience and time-decay treatments were significant (Table 2, 2^nd^ to fifth sections). Other than modulating the importance of contest experience to fighting behaviour, pre-contest KT also appeared to have a direct positive association with the focal individuals’ probability of behaving aggressively and winning. The importance of the experience effect on both the probability of behaving aggressively and the probability of winning was dependent on pre-contest T and KT as was its longevity. (There were significant ‘experience×pre-contest level’, ‘time-decay×pre-contest level’ and/or ‘experience×time-decay×pre-contest level’ effects, see Table 2). The importance of the effect of experience on the probability of behaving aggressively also depended on F (significant ‘experience×time-decay×pre-contest F’ effects, Table 2). There was no similar relationship between F, experience and the probability of winning. E2 did not appear to have much influence on the experience effects as none of the interaction effects between pre-contest E2, contest experience and time-decay reached significance (Table 2, 5^th^ section).

**Figure 3:**
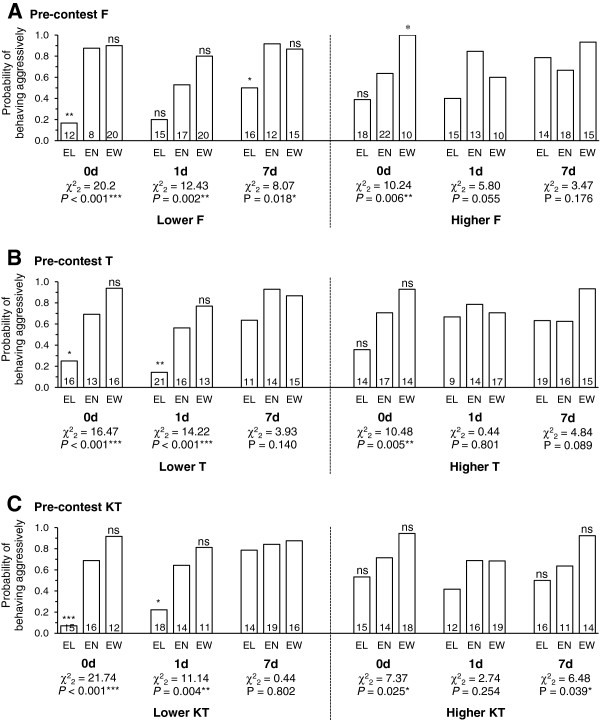
**The influence of winning/losing experience on aggressiveness for individuals with lower/higher hormone levels.** The decay of the effect of experience on the probability of behaving aggressively for focal individuals with lower (< median) and higher (≥ median) levels of **(A)** pre-contest F, **(B)** pre-contest T and **(C)** pre-contest KT. Pearson’s χ^2^ tests were used to determine the significance of overall experience effects for each time-decay treatment/hormone-level group. For treatments with significant overall experience effects, Fisher’s exact tests (2-tailed) were then used to test the significance of loser and winner effects separately by comparing the probabilities for the EL and the EW fish, respectively, to that for the EN fish (shown on tops of EL and EW bars, respectively). The sample size for each bar is presented on the bottom of the bar. ns *P* > 0.05; * P ≤ 0.05; ** *P* ≤ 0.01; *** *P* ≤ 0.001.

To illustrate the complex relationships between the importance and longevity of experience effects and pre-contest F, T and KT more clearly, we grouped focal individuals into those having lower (< median) or higher (≥ median) levels of the hormones and showed how the experience effects changed over time for these two groups of individuals. Figures 3 (probability of behaving aggressively) and 4 (probability of winning) show similar trends, i.e., for experience effects to be more significant and last longer for focal individuals with lower levels of F, T, or KT. These trends appeared to be caused by the focal individuals with lower levels of hormones showing stronger loser effects.

**Figure 4:**
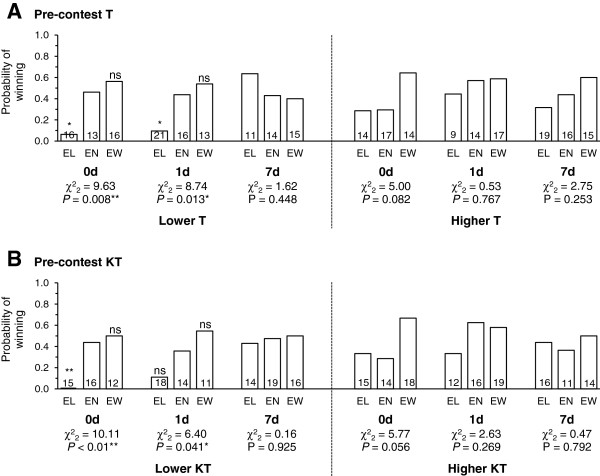
**Influence of winning/losing experience on winning probability for individuals with lower/higher hormone levels.** The decay of the effect of experience on the probability of winning for focal individuals with lower (< median) and higher (≥ median) levels of **(A)** pre-contest T and **(B)** pre-contest KT. This analysis was not carried out for individuals with lower and higher levels of pre-contest F as F had no significant influence on winning probability (Table 2). Pearson’s χ^2^ tests were used to determine the significance of overall experience effects for each time-decay treatment/hormone-level group. For the treatments with significant overall experience effects, Fisher’s exact tests (2-tailed) were then used to test the significance of loser and winner effects separately by comparing the probabilities for the EL and the EW fish, respectively, to that for the EN fish (shown on tops of EL and EW bars, respectively). The sample size for each bar is presented on the bottom of the bar. ns *P* > 0.05; * P ≤ 0.05; ** *P* ≤ 0.01.

### Differences in the levels of hormones between the winners and losers of the size-matched contests

**Figure 5:**
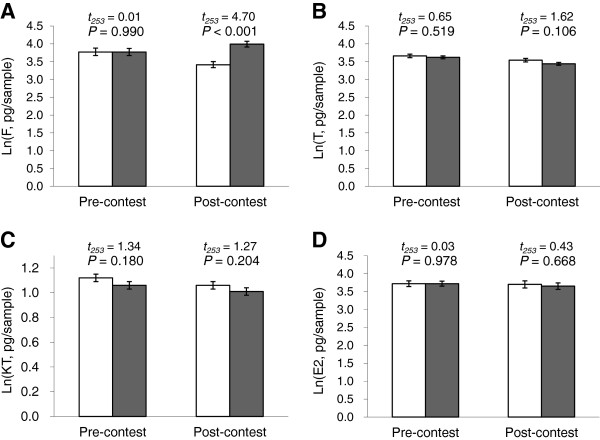
**Pre-contest and post-contest hormone levels for focal individuals that won and that lost size-matched contests.** Levels of **(A)** F, **(B)** T, **(C)** KT, and **(D)** E2 (ln transformed, mean ± SE) of the focal individuals that won (clear bars; *N* = 113) and lost (shaded bars; *N* = 142) the size-matched contests.

Focal individuals that lost to their size-matched opponents had significantly higher levels of post-contest F than those that won (Figure 5A), despite the fact that F levels of these two groups of focal individuals did not differ prior to the contests. Focal individuals that won or lost the contests with a size-matched opponent did not differ significantly in the levels of T, KT or E2 prior to or after the contests (Figure 5B-D).

**Figure 6:**
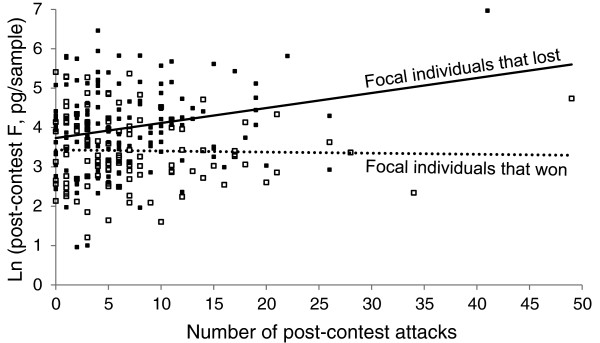
**The influence of post-contest attacks on post-contest F levels.** Post-contest F levels (ln transformed) for the focal individuals that won (clear squares) and lost (filled squares) to their size-matched opponents, where winners delivered different numbers of post-retreat attacks to the losers. The broken line shows the relationship between the focal individuals’ post-contest F levels and the number of attacks they delivered to their defeated opponents (for the focal individuals that won), and the solid line shows the relationship between the focal individuals’ post-contest F levels and the number of attacks they received from their victorious opponents (for the focal individuals that lost).

Since winning and losing size-matched contests gave rise to a significant difference in levels of F, but winning and losing experience training did not, we tested the hypothesis that this difference arose because of a difference in procedure: in the experience training, the focal individuals were separated from their smaller or larger trainers as soon as the loser had retreated, but in the size-matched contests the focal individuals were allowed a further five minutes of post-contest interaction with their matched opponents, during which time the winner sometimes continued to attack the loser. We therefore used a multiple linear regression model (overall model significance: *F*_3,251_ = 10.53, *P* < 0.001) to examine simultaneously whether the focal individual’s post-contest F was modulated by the number of post-contest attacks (*F*_1,251_ = 0.05, *P* = 0.821), by the focal individual winning or losing the contest with its size-matched naïve opponent (*F*_1,251_ = 3.03, *P* = 0.083), and the interaction between the two (*F*_1,251_ = 5.35, *P* = 0.022). The significant interaction effect arose because the relationships between post-contest F and post-contest attacks were different for focal individuals that won and that lost the contests with their matched opponents; the post-contest F of those that won was not significantly related to the number of attacks they delivered to the size-matched opponents they had already defeated (slope ± SE = -0.00 ± 0.01, *F*_1,251_ = 0.05, *P* = 0.821), while the post-contest F of those that lost increased with the number of attacks they received from the opponents that had defeated them (slope ± SE = 0.04 ± 0.01, *F*_1,251_ = 8.88, *P* = 0.003) (Figure 6). These trends and the marginally non-significant effect of the focal individual winning or losing the contests are consistent with the hypothesis that the difference in post-contest F between focal individuals that won or lost to their size-matched opponents resulted primarily from the post-contest attacks rather than from winning and losing per se.

## Discussion

This study showed that an individual’s propensity to adjust contest decisions after wins and losses depends on its hormonal state: individuals with lower levels of F, T and KT were more receptive to the influence of recent contest experiences and retained the influence for longer than individuals with higher levels of the hormones. In this study, KT was the only hormone that had a significant positive correlation with the probabilities of behaving aggressively and of winning the size-matched contests. In past studies, however, T correlated positively with winning contests [23] and both T and F correlated positively with the fish’s aggressiveness towards its mirror image [22]. Taking together the findings that individuals with a losing experience one month previously [9] and those with lower levels of F, T or KT (this study) were more prone to changing contest behaviours after a recent win or loss, the evidence seems to indicate that, in this fish, less aggressive individuals (because of previous losing experiences and/or lower levels of aggression-related hormones) exhibit higher flexibility in contest strategies, i.e., have a higher propensity to adjust contest decisions based on the outcomes of recent fights. In the field, individuals that behave submissively probably have worse than average competitive abilities, and the costs to them of engaging in contests are more dependent on the fighting ability of the other individuals in their local populations. It therefore pays for them to monitor the fighting ability of the individuals in their local populations more closely. This hypothesis suggests that the asymmetry in the value of new information between individuals with better and worse fighting ability should be positively associated with population turnover and the degree of heterogeneity between populations in the composition of individuals’ fighting ability, a prediction which awaits examination. Our study also showed that individuals with lower levels of the hormones responded more strongly to losing than to winning experiences. A losing experience probably confirms a weaker individual’s initial perception of its worse fighting ability, and reduces the uncertainty in fighting costs more than a winning experience. The cost of losing may also be greater than the benefit of winning [3], especially for weaker individuals, less able to recover from exhaustion or injury. The differences in both uncertainty reduction and cost/benefit could cause these weaker individuals to be more conservative in modifying contest behaviour after wins than after losses.

Many studies have explored how individuals of different dominance status differ in their receptivity to new information and their readiness to modify behaviour in response to it. Although these studies reach different conclusions, it seems that individuals of different dominance status are sensitive to different types of information (see [9] for a brief discussion): dominant individuals appear to be more receptive to information about their physical environment and to be better at spatial learning and food-reward associative learning [26-29], while subordinate individuals tend to be more sensitive to information associated with social learning and predation risks [30,31]. For instance, dominant chickadees (*Poecile gambeli*) showed better spatial memory than subordinates in tasks relating to recovery of hidden food [26], while subordinate crabs (*Chasmagnathus granulatus*) showed higher memory retention than dominants in tests involving visual danger stimuli [30]. Individuals of *K. marmoratus* that received a forced winning experience not only behaved more aggressively but also performed better in shelter-related spatial learning tasks than those that received a forced losing experience [22]. Considering together the findings that individuals receiving a losing experience one month previously [9] and individuals with lower levels of the steroid hormones (present study) were both more sensitive to information generated from recent contest experiences, the fish’s behaviour is consistent with the hypothesis that individuals with different dominance statuses are receptive to different types of information. More effort will be needed to understand how common it is for different groups of individuals, or the same individuals in different situations, to vary in their propensity to change behaviour when exposed to various types of information. Effort will also be needed to understand its biological importance.

An individual’s propensity to modify contest behaviour after wins or losses has been shown to be a flexible trait: it can be influenced by extrinsic factors such as the contest experience forced on the individual one month previously [9] or the contest environment (as shown in California mice: [8]). This study further showed that the propensity is also modulated by internal factors such as an individual’s hormonal state. An individual’s readiness to alter contest strategies as a result of the outcome of a recent contest depended on both its hormonal state prior to receiving the experience and the changes in the levels of the hormones in the period between the first hormone measurements and the time of the new contest. The changes in hormone levels, however, did not vary systematically with either the experience treatment or time lapse, which ranged from 3 to 4 and 10 days for 0d, 1d and 7d time-decay treatments, respectively. Many factors could have contributed to the variation in the changes, including both the natural fluctuation in hormone levels (independent of experimental procedures) and individual hormonal responses to disturbance independent of treatment type. Because pre-experience levels and the changes in the levels together make up pre-contest hormones levels, the results of this study indicated that how an individual utilizes information derived from previous contests depended on its hormonal state at the time of the new contest, which suggests that individuals monitor their physiological conditions closely. It is not entirely clear which factors dictate whether and how individuals use information from recent contest experience to modify their subsequent contest decisions, because of the scarcity of relevant studies. If the outcomes of recent fights provide individuals with information about how their fighting ability compares to that of the others in the population [5,6], factors that relate to an individual’s ability to defeat an opponent and/or the amount of information the individual has already accumulated are probably important to whether and how individuals utilize information from recent contest experiences. For instance, an individual that is at its peak growth rate might retain information from a previous fight for less time than an individual that is no longer growing [3]. On the other hand, based on the assumption that individuals lack direct knowledge of their own fighting ability and that of their opponents, Fawcett and Johnstone’s model [32] predicted that young and naïve individuals should show pronounced loser effects while older individuals who have a better idea of their own fighting ability should be more responsive to victories than losses. All these predictions still remain untested.

The results of this study showed that although the pre-designated winning or losing experiences brought about changes in *K. marmoratus*’*s* contest strategies, they did not significantly affect the fishes’ levels of F, T, KT or E2. Hormone titres therefore were probably not the primary proximate mechanisms mediating the behavioural changes after contest experiences, consistent with the findings of Chang et al. [22]. The fact that F, T or KT did not change after winning or losing is not unique to *K*. *marmoratus*. Individuals of different dominance status (winners, losers, control) in male African cichlids (*Tilapia zillii*) also did not differ in the levels of post-contest T or KT [33] and individuals with different dominance statuses in male convict cichlids (*Amatitlania nigrofasciata*) did not differ in the levels of post-contest T, KT or F [34]. However, these results are intriguing, because these hormones have been repeatedly shown to have close relationships with this fish’s contest behaviour and performance [22,23] as they have in many other fish [24,25,35] and other vertebrates [13-15,18]. The implication of this study’s results is that the winner and loser effects in this fish are mediated by some physiological mechanisms that cause changes in contest behaviour without affecting the levels of these hormones, despite the fact these steroid hormones have close relationships with the fish’s contest behaviour and performance. Possible candidates include changes in gene expression patterns, steroid receptor densities and the secretion of neuromodulators, all of which have been shown to have close associations with contest behaviour and performance. For example, relative to subordinates, dominant African cichlid (*Astatotilapia burtoni*) showed elevated androgen receptor mRNA expression in the anterior portion of the brain [36]. In Mozambique tilapia (*Oreochromis mossambicus*), contest winners whose androgen receptors were blocked failed to display a winner effect while untreated winners did [37]. And, compared with the saline controls, intermediate doses of AVT increased aggressiveness levels in male damselfish (*Stegastes leucostictus*) [38]. The involvement of these physiological mechanisms in modulating the winner and/or loser effects in *K*. *marmoratus* requires further investigation.

The focal individuals that won and that lost against their size-matched opponents differed significantly in post-contest F levels despite the fact that F levels remained relatively unchanged after the forced winning or losing experiences. Further analyses showed that the differences in post-contest F between the focal individuals that won and that lost the size-matched contests were probably caused by the post-retreat attacks delivered to the focal individuals that lost – those that received more attacks had higher levels of post-contest F. The results of this study therefore indicate that subordinate status itself does not cause elevated F in this fish - a similar conclusion to that reported in convict cichlids (*A*. *nigrofasciata*) [34,39]. However, being subjected to attacks does cause an elevation in losers’ F levels. A previous study showed that baseline F levels in *K*. *marmoratus* correlated positively with baseline T levels as well as the fish’s aggressiveness towards a mirror image [22]. That aggressive individuals have higher pre-fight corticosteroid levels than nonaggressive individuals was also discovered in the lizard *Anolis carolinensis*[40]. And, after 40 min of social interaction, subordinate lizards’ corticosterone levels were elevated and were higher than those of dominants [41]. Overall, the role of corticosteroids in contest decisions is complex and is associated with both aggression and stress responses [13], as we have also discovered in *K*. *marmoratus*. Further studies, including studies manipulating the levels of the hormone, might help us gain more insights into its importance in influencing the fish’s contest behaviour.

## Conclusions

This study showed that individuals of *K*. *marmoratus* with lower levels of F, T and KT are more receptive to the influence of recent contest experiences and retain the influence for longer than individuals with higher levels of the hormones. In particular, fish with lower levels of the hormones showed a stronger and more long-lasting loser effect than those with higher levels of the hormones. Levels of these hormones in the fish are positively correlated with its aggressiveness and/or probability of winning contests, which indicates that individuals with hormonal profiles corresponding to subordination and a reduced likelihood of winning are more inclined to adjust contest strategies in the light of new information. This study also found that, although the fish’s contest behaviour is closely associated with F, T and KT levels, hormone titres are not the main physical mechanism mediating the winner and loser effects in this fish: the experience training led to winner/loser effects but not to significant changes in hormone levels. In looking for the physical mechanisms underlying the winner and loser effects in this fish, we may therefore want to study other candidates known to influence behaviour and aggressiveness such as changes in gene expression patterns, steroid receptor densities and the secretion of neuromodulators.

## Materials and methods

### Study organism

*Kryptolebias marmoratus* is an internally self-fertilizing hermaphroditic fish [42], living in mangrove swamps from coastal regions of Brazil and eastern Central America, throughout the Caribbean to central Florida [43]. Natural populations mainly consist of isogenic homozygous hermaphrodites with very low incidence (< 1%) of males, although an out-crossing heterozygous population with approximately 20% males was discovered in Twin Cays, Belize [44]. This study used five strains of *K*. *marmoratus* from various geographical areas (DAN2K: Dangria, Belize; HON9: Utila, Honduras; RHL: San Salvador, Bahamas; SLC8E: St. Lucie County, FL, USA; VOL: Volusia County, Florida, USA), which were F3 to F6 generations of fish originally collected from the field by Dr. D. Scott Taylor. Fish were isolated within a week of hatching in a laboratory at National Taiwan Normal University, given a unique identification code and kept alone in a 13 × 13 × 9 × cm^3^ translucent polypropylene container filled with 550 ml 25 ppt synthetic sea water (Instant Ocean™ powder). Fish were kept at 25 ± 2°C on a 14:10-h photoperiod and fed newly hatched brine shrimp (Artemia) nauplii daily. Containers were cleaned and water replaced every 2 weeks.

Experiments were conducted in accordance with a protocol approved by The Animal Care and Use Committee of National Taiwan Normal University (permit #96016).

### Experimental design and procedures

We used a 3 × 3 factorial design to examine whether the significance and longevity of the winner and loser effects in this fish were dependent on the levels of F, T, KT and E2: three experience treatments (three consecutive winning experiences: EW, three losing experiences: EL, and no recent experience: EN) and three time-decay treatments (0 day: 0d, one day: 1d, and 7 days: 7d), for a total of 9 treatment combinations. The focal individuals were given the same contest experience three times to enhance the effects of the experiences: the effects from multiple contest experiences in this fish are cumulative [45]. The influence of one winning/losing experience is strongest shortly after the completion of the experience, decaying over time and becoming undetectable after 2 to 4 days [46].

All fish used in this study had been re-isolated for at least one month after use in previous studies, and contest pairs were matched for their last contest outcome (i.e., previous winners with previous winners and previous losers with previous losers). Contest pairs were also matched for strain type, body size (difference in body length ≤ 1 mm) and age (difference in age ≤ 15 days). Matched pairs were randomly assigned to the 9 treatments. One individual of a matched pair was randomly chosen to be the focal individual and subjected to experience training (EW, EL or EN) and hormone measurements. The other individual served as the matched opponent in the final staged contest (size-matched contest) and was not subjected to experience training or hormone measurements. Each fish was used only once in this study.

On Day 1, at 1100 h, we removed focal individuals from their maintenance containers to collect water samples for baseline (pre-experience) hormones and returned them to their containers after the procedures. On Days 2 to 4, starting at 1000 h, focal individuals were given their pre-designated winning, losing or no contest experiences - one experience per day for three consecutive days. On Day 4, Day 5 and Day 11, at 1100 h, water samples were collected from focal individuals assigned to 0d, 1d and 7d time-decay treatments, respectively, for pre-contest hormones. Afterwards, we placed the focal individuals and their size-matched opponents in the containers for their size-matched contests. (See below for procedures.) Immediately after the contests were completed, water samples were again collected from the focal individuals for post-contest hormones.

### Collection, extraction and assay of hormones

For each hormone collection, each focal individual was placed for 1 h in its own 400 ml glass beaker filled with 200 ml clean 25 ppt synthetic seawater housed inside an individual translucent plastic container. Water was then removed from the beaker with a vacuum pump and passed through a C18 solid-phase column (Lichrolut RP-18, 500 mg, 3.0 ml; Merck) fitted to a 12-port manifold to extract hormones. Before use, the columns were first primed with 2 consecutive washes with 2 ml methanol followed by 2 consecutive washes with 2 ml distilled water. After use, the columns were purged of sea salt with 2 consecutive 2 ml washes of distilled water and stored at -80°C until further processing. Freeze storage of water samples and columns has been determined not to impact steroid concentrations [47].

Hormones were eluted from the columns by 2 × 2 ml ethyl acetate washes. The eluted solvent was evaporated using vacuum centrifuge (Savant SpeedVac^®^ Systems). The resulting hormone residue was re-suspended in 800 ml of enzyme-immunoassay (EIA) buffer supplied with the Cayman Chemicals Inc. EIA kits, and the samples were stored at -20°C until assay. Cayman Chemicals Inc. EIA kits were used for all hormones (F, T, KT and E2), following the manufacturer’s recommended procedures. Plates were read at 405 nm on a BioMek microplate reader. Assays of F, T and KT in *K*. *marmoratus* using Cayman Chemicals Inc. EIA kits has been previously validated by Earley & Hsu [23]. E2 was validated by serially diluting pooled water-borne hormone extract from 50 non-experimental animals representing the five *K*. *marmoratus* strains. The serial dilution curve was parallel to the standard curve (comparison of slopes: t_12_ = 0.01, p = 0.99). All the data on hormone levels are presented as pg/ml. Intra-assay coefficients of variation ranged from 2.1-5.3% for F (median: 3.7%; N = 30 plates), from 0.9%-26.4% for T (median: 4.4%; N = 30 plates), from 1.5-7.2% for KT (median: 3.6%; N = 30 plates) and from 2.1-12.9% for E2 (median: 6.3%; N = 30 plates). The inter-assay coefficient of variation was 7.9% for F, 13.2% for T, 10.5% for KT and 14.4% for E2. The sensitivities of the assays (range: plates 1-30) were as follows: F (2.9-6.9 pg/ml); T (1.5-5.1 pg/ml); KT (1.71-2.44 pg/ml); E2 (11.37-30.41 pg/ml).

### Providing winning, losing or no-contest experiences

To ensure that the focal individuals received winning or losing experiences as determined, we fought them against much smaller/larger (difference > 2 mm) fish that had lost/won several fights against similar-sized opponents. For experience training, a focal individual and a smaller/larger trainer fish were each placed in one of the two equal-sized, symmetrical compartments (randomly assigned) of a standard aquarium (12 × 8 × 20 cm, containing water 16 cm deep and 2 cm of gravel) divided by an opaque partition. After 15-min acclimatisation the partition was removed to allow the fish to interact. A winning experience was completed when the smaller trainer fish retreated from the focal individual’s display/attack and quickly swam away. A losing experience was completed when the focal individual retreated from a display/attack by the larger trainer fish and quickly swam away. Experiment individuals acquired their pre-designated experiences quickly (median = 37 s, 19 s, 17 s for the 1^st^, 2^nd^ and 3^rd^ winning experience, respectively; median = 30 s, 16 s, and 22 s for the 1^st^, 2^nd^ and 3^rd^ losing experience, respectively), and the partition was reinserted to separate the two fish immediately afterwards. Three different smaller/larger trainer fish were used to administer the three winning/losing experience-trainings to avoid the possible complication of individual recognition on experience effects. Fish assigned to receive no (EN) experience were treated exactly as above, with procedures synchronised to those assigned EW or EL treatments and trained at the same time, except with no opponent in the other compartment.

### Staging size-matched contests

The focal individuals and their matched opponents were each placed in one of the two compartments (randomly assigned) of a standard aquarium separated by an opaque partition to acclimatize for 2 h. After the partition was removed, the fish usually oriented and moved towards each other. After a few bouts of mutual displays, one fish sometimes retreated. If not, one fish launched a first attack by swimming rapidly towards and pushing against or biting its opponent and was the attack initiator. Sometimes the fish receiving the first attack retreated; otherwise its opponent responded with attacks. The individual that first persistently retreated from its opponent’s displays/attacks for 5 min without retaliating was the contest loser, and its opponent the winner. If no obvious winner/loser was observed in 1 h, the contest was terminated and classified as “unresolved”.

### Statistical analyses

We staged a total of 270 contests, 30 for each of the nine treatment combinations, evenly distributed over the 5 strains of the fish. We first used multiple linear regression models to examine whether focal individuals’ pre-contest hormones varied with their experience × time-decay treatments, controlling for pre-experience hormone level and strain type.

We then used multiple logistic regressions to explore whether an individual’s behavioural responses to the size-matched opponent after being exposed to different experience × time-decay treatments depended on its hormonal state, controlling for strain type. The behavioural responses examined were whether or not the focal individual was aggressive and whether or not the focal individual won in the size-matched contests. If a focal individual initiated attacks or retaliated with attacks when attacked, it was deemed to be aggressive. Significant interaction effects between contest experience and hormone levels on the behavioural responses measured would indicate a dependence of winner and/or loser effects on hormonal states. We constructed two sets of regression models, one using just pre-contest hormone levels to represent the fishes’ hormonal states and a second using both pre-experience hormone levels and the change from pre-experience to pre-contest levels and used a likelihood ratio test to determine whether the more complex model performed significantly better in explaining the variability in the behavioural responses.

Finally, we used a multiple linear regression model to determine how focal individuals’ post-contest F levels varied with (a) the number of attacks delivered after the resolution of the contest by the winner to the loser, (b) the focal individual’s result in the contest (winning or losing) and (c) the interaction between (a) and (b). Focal individuals that won delivered attacks to their defeated size-matched opponents; those that lost received attacks from their victorious size-matched opponents. Each contest pair therefore only contributed one data point to this analysis, as in all the other analyses in this study.

Hormone levels were natural-log transformed. JMP (v. 5.0.1 SAS Institute Inc., Cary, NC, USA), a commercial statistical package, was used for the statistical analyses.

## Competing interests

The authors declare that they have no competing interests.

## Authors’ contributions

YH conceived of, designed and supervised the experiments and contributed to statistical analysis and the manuscript. RLE contributed to the design of the experiments, hormone assay and the manuscript. CKL and IHL conducted the most significant part of the experiments and contributed to data analysis and an early draft of the manuscript. SCW contributed to hormone assay and the early draft of the manuscript. All authors read and approved the final manuscript.

## Supplementary Material

Additional file 1 **The importance of hormonal states to the influence of contest experience - the full models.** The models presented here are the “full” versions of the models presented in Table 2. The pre-contest hormone levels in Table 2 are here divided into two: pre-experience hormone levels and Δ = pre-contest levels – pre-experience levels The importance of these two levels to the influence of contest experiences on the probability of behaving aggressively and winning contests were tested. (*N* = 270, *LR*χ^*2*^: likelihood ratio χ^*2*^, *: *P* ≤ 0.05, Exp: Experience, Time: Time-decay). Click here for file
