# Supplementary material for: Winner and loser effects are modulated by hormonal states
Source: Front Zool. 2013 Feb 11;10:6. doi: 10.1186/1742-9994-10-6 (PMC3598835; doi:10.1186/1742-9994-10-6)
Supplement: Additional file 1 — The importance of hormonal states to the influence of contest experience - the full models. The models presented here are the “full” versions of the models presented in Table 2. The pre-contest hormone levels in Table 2 are here divided into two: pre-experience hormone levels and Δ = pre-contest levels – pre-experience levels The importance of these two levels to the influence of contest experiences on the probability of behaving aggressively and winning contests were tested. (N = 270, LRχ2: likelihood ratio χ2, *: P ≤ 0.05, Exp: Experience, Time: Time-decay). [file 1742-9994-10-6-S1.docx]

**Additional File 1:**

**The importance of hormonal states to the influence of contest experience - the full models.**

The models presented here are the “full” version of the models presented in Table 2. The pre-contest hormone levels in table 2 are here divided into two levels, pre-experience hormone levels and Δ = pre-contest levels – pre-experience levels. The importance of these two levels to the influence of contest experiences on the probability of behaving aggressively and winning contests were tested. (*N* = 270, *LRχ^2^*: likelihood ratio *χ^2^*, *: *P* ≤ 0.05, Exp: Experience, Time: Time-decay)

|  |  | **Behaving aggressively** | | |  | **Winning contests** | | |  |
| --- | --- | --- | --- | --- | --- | --- | --- | --- | --- |
| ***Variable*** | ***df*** | ***b ±SE*** | ***LRχ^2^*** | ***P*** |  | ***b ±SE*** | ***LRχ^2^*** | ***P*** |  |
| Pre-Exp F | 1 | 0.16±0.29 | 0.33 | 0.566 |  | -0.16±0.17 | 0.88 | 0.349 |  |
| Exp×Pre-Exp F | 2 |  | 6.00 | 0.050 | * |  | 1.54 | 0.463 |  |
| Time×Pre-Exp F | 2 |  | 6.01 | 0.050 | * |  | 2.64 | 0.267 |  |
| Exp×Time×Pre-Exp F | 4 |  | 11.76 | 0.019 | * |  | 5.85 | 0.211 |  |
| ΔF | 1 | 0.17±0.21 | 0.66 | 0.417 |  | -0.01±0.14 | 0.01 | 0.941 |  |
| Exp×ΔF | 2 |  | 8.56 | 0.014 | * |  | 0.50 | 0.780 |  |
| Time×ΔF | 2 |  | 2.05 | 0.360 |  |  | 0.34 | 0.844 |  |
| Exp×Time×ΔF | 4 |  | 12.74 | 0.013 | * |  | 0.65 | 0. 958 |  |
| Pre-Exp T | 1 | 1.13±0.69 | 3.03 | 0.082 |  | 0.16±0.43 | 0.15 | 0.703 |  |
| Exp×Pre-Exp T | 2 |  | 12.52 | 0.002 | * |  | 0.99 | 0.610 |  |
| Time×Pre-Exp T | 2 |  | 1.75 | 0.417 |  |  | 4.13 | 0.127 |  |
| Exp×Time×Pre-Exp T | 4 |  | 11.87 | 0.018 | * |  | 13.38 | 0.010 | * |
| ΔT | 1 | 0.47±0.59 | 0.64 | 0.424 |  | 0.06±0.42 | 0.02 | 0.890 |  |
| Exp×ΔT | 2 |  | 7.44 | 0.024 | * |  | 1.35 | 0.510 |  |
| Time×ΔT | 2 |  | 1.03 | 0.598 |  |  | 1.47 | 0.479 |  |
| Exp×Time×ΔT | 4 |  | 10.24 | 0.037 | * |  | 8.09 | 0.088 |  |
| Pre-Exp KT | 1 | 1.41±0.74 | 3.89 | 0.049 | * | 1.79±0.78 | 6.58 | 0.010 | * |
| Exp×Pre-Exp KT | 2 |  | 7.99 | 0.018 | * |  | 7.70 | 0.021 | * |
| Time×Pre-Exp KT | 2 |  | 9.14 | 0.010 | * |  | 7.79 | 0.020 | * |
| Exp×Time×Pre-Exp KT | 4 |  | 8.36 | 0.079 |  |  | 12.47 | 0.014 | * |
| ΔKT | 1 | 1.51±0.77 | 4.04 | 0.044 | * | 1.48±0.80 | 3.92 | 0.048 | * |
| Exp×ΔKT | 2 |  | 8.99 | 0.011 | * |  | 7.88 | 0.020 | * |
| Time×ΔKT | 2 |  | 7.55 | 0.023 | * |  | 1.15 | 0.563 |  |
| Exp×Time×ΔKT | 4 |  | 1.11 | 0.893 |  |  | 6.95 | 0.139 |  |
| Pre-Exp E2 | 1 | 0.23±0.33 | 0.49 | 0.484 |  | -0.15±0.25 | 0.38 | 0.540 |  |
| Exp×Pre-Exp E2 | 2 |  | 1.06 | 0.590 |  |  | 0.35 | 0.840 |  |
| Time×Pre-Exp E2 | 2 |  | 2.35 | 0.309 |  |  | 4.65 | 0.098 |  |
| Exp×Time×Pre-Exp E2 | 4 |  | 9.69 | 0.046 | * |  | 8.49 | 0.075 |  |
| ΔE2 | 1 | -0.20±0.33 | 0.38 | 0.538 |  | 0.01±0.22 | 0.00 | 0.977 |  |
| Exp×ΔE2 | 2 |  | 3.89 | 0.143 |  |  | 0.97 | 0.616 |  |
| Time×ΔE2 | 2 |  | 2.84 | 0.242 |  |  | 0.47 | 0.791 |  |
| Exp×Time×ΔE2 | 4 |  | 8.24 | 0.083 |  |  | 7.74 | 0.102 |  |
